# Green Technologies for Persimmon By-Products Revalorisation as Sustainable Sources of Dietary Fibre and Antioxidants for Functional Beverages Development

**DOI:** 10.3390/antiox12051085

**Published:** 2023-05-12

**Authors:** Julio Salazar-Bermeo, Bryan Moreno-Chamba, Rosa Heredia-Hortigüela, Victoria Lizama, María Concepción Martínez-Madrid, Domingo Saura, Manuel Valero, Madalina Neacsu, Nuria Martí

**Affiliations:** 1 Instituto de Investigación, Desarrollo e Innovación en Biotecnología Sanitaria de Elche (IDiBE), Universidad Miguel Hernández de Elche, 03202 Alicante, Spain; julio.salazar@goumh.umh.es (J.S.-B.); bryan.morenoc@umh.es (B.M.-C.); rosa.heredia@goumh.umh.es (R.H.-H.); c.martinez@umh.es (M.C.M.-M.); dsaura@umh.es (D.S.); m.valero@umh.es (M.V.); 2 Instituto de Ingeniería de Alimentos para el Desarrollo, Universitat Politècnica de València, Avenida Fausto Elio s/n, Edificio 8E, Acceso F Planta 0, 46022 Valencia, Spain; vlizama@tal.upv.es; 3 The Rowett Institute, University of Aberdeen, Aberdeen AB25 2ZD, UK; m.neacsu@abdn.ac.uk

**Keywords:** functional beverages, *Diospyros kaki*, ultrasound, natural eutectic, polysaccharides, by-products, dietary fibre, antioxidant bioactives

## Abstract

The use of green technologies such as ultrasound and natural deep eutectic solvents (NADES) for revalorisation of food and agricultural by-products represents a sustainable way to tackle waste and promote a healthier environment while delivering much-needed functional food ingredients for an increasingly unhealthy population. The processing of persimmon (*Diospyros kaki* Thunb.) generates large amounts of by-products rich in fibre-bound bioactive phytochemicals. This paper assessed the extractability of bioactive compounds through NADES and the functional properties of the persimmon polysaccharide-rich by-products to evaluate their suitability to be used as functional ingredients in commercial beverages. Although higher amounts of carotenoids and polyphenols were extracted after eutectic treatment vs. conventional extraction (*p* < 0.05), the fibre-bound bioactives remained abundant (*p* < 0.001) in the resulting persimmon pulp by-product (PPBP) and persimmon pulp dietary fibre (PPDF), showing also a strong antioxidant activity (DPPH^•^, ABTS^•+^ assays) and an improved digestibility and fibre fermentability. The main structural components of PPBP and PPDF are cellulose, hemicellulose and pectin. PPDF-added dairy-based drink showed more than 50% of preference over the control among panellists and similar acceptability scores to the commercial ones. Persimmon pulp by-products represent sustainable source of dietary fibre and bioactives and are suitable candidates to develop functional ingredients for food industry applications.

## 1. Introduction

Food production and agro-industrial processing to feed a growing population generate high levels of by-products (such as peels, pomace, hulls and leaves) [1], with a significant negative environmental impact. The losses or wastage of food represent between 30 and 50% of all food produced (approx. 220 million tons of food wasted every year), the equivalent of the entire net food production of sub-Saharan Africa [2].

Valorising by-products for food is an actual topic, and research could contribute to circular nutrition and agricultural sustainability. Additionally, the development of nutraceuticals, supplements, and functional food ingredients could help tackle global dietary deficiency/malnutrition and diseases by delivering important nutrients such as dietary fibre, protein, minerals and bioactive phytochemicals. Food fortification [3] can be utilised from agricultural by-products, as seen in the initiative to address dietary deficiencies [4] in Europe and the USA.

Among food products, beverages are a very popular fast food, with only a few containing functional food ingredients, such as dietary fibre. The addition of polysaccharides as ingredients for the preparation of beverages can address nutritional insufficiencies and recommendations. The enrichment of beverages with dietary fibre could therefore deliver the daily fibre recommendations (25–32 g/day for women, 30–35 g/day for men). A diet rich in fibre has numerous health benefits; consuming over 25 g/day has been related to a reduction in the risk of coronary heart disease, regulation of metabolic syndromes such as obesity and type 2 diabetes, modulation of complex communities in the gut microbiome, increased faecal bulk, and enhancement of the immune response [5,6,7].

To revalorise the dietary fibre from food by-products, the polysaccharides need to be treated prior to use, not only for their functional properties and food safety but also to meet sensorial requirements. The addition of dietary polysaccharides to beverages presents challenges in maintaining their suspension without negatively impacting the sensory profile of the product. Food by-product processing involves extensive and harsh operating conditions and the use of solvents, which consequently require treatment of residual effluents. Ultrasonic treatment represents a green alternative to treating and processing food by-products and obtaining dietary fibre (polysaccharide)-rich fractions. The rotation and collision of molecules by the ultrasonic treatment produce the rupture of walls and structures within the polymer. In addition, a new class of more sustainable solvents, known as natural deep eutectic solvents (NADES), is being developed. NADES are formed as a result of hydrogen bonding between receptor and donor compounds, together with other intermolecular interactions such as van der Waals and electrostatic forces [8]. The pairing of ultrasonic technology with NADES is a new area of development. This technology extracts higher amounts of compounds from the polysaccharide matrix and purifies polysaccharides while increasing the bioavailability of fibre-bound phytochemicals.

Among fruit-based by-products, persimmon juice by-products are a rich source of bioactive phytochemicals such as carotenoids (lutein, zeaxanthin, β-cryptoxanthin, β-carotene, α-carotene, and lycopene), polyphenols (gallic acid, fumaric acid, epigallocatechin, and catechin), and monosaccharides (glucose and fructose) [9,10,11] with health properties associated with persimmon’s composition [12]. Pulp waste and peels are two of the main by-products generated from persimmon juice processing, which is still an unexplored source of bound bioactive phytochemicals.

There is a plethora of information related to the composition of persimmon fruit polysaccharides. Although, monosaccharides in peel and extracted pectin have been reported as D-rhamnose (Rha), L-fucose (Fuc), arabinose (Ara), galacturonic acid (GalA), D-xylose (Xyl), D-mannose (Man), D-galactose (Gal), and D-glucose (Glu), and the persimmon dietary fibre functional properties have been assessed [13,14,15], further investigations are necessary to understand the potential of persimmon by-product polysaccharides to be revalorised for food consumption.

The present work aims to produce fibre-rich food ingredients from persimmon by-products using ultrasonic treatment (US) in NADES. Functional beverages were consequently produced by the incorporation of the treated polysaccharides (fibre-rich fractions). The tannin, flavonoid, carotenoid, and polyphenol removal efficiency by the eutectic extraction was also assessed. Results were studied and examined in terms of available eutectic extract and fibre-bound phytochemical content and composition. The composition and technological (physical) properties of fibre-rich fractions were assessed, and finally, the sensorial profile of the functional beverages containing the persimmon fibre-rich fractions was assessed.

## 2. Materials and Methods

### 2.1. Reagents

Acetone (99.9%), glacial acetic acid (99.8%), acetonitrile (99.9%), absolute ethanol, methanol (LC-MS grade), HCl (37%), chloroform, and sodium hydroxide were obtained from PanReac (Barcelona, Spain). Folin–Ciocalteu reagent, ABTS^•+^ (2,2′-azino-bis(3-ethylbenzothiazoline-6-sulphonic acid), DPPH^•^ (1,1-diphenyl-2-picrylhydrazyl), gallic acid, quercetin, cyanidin-3 glucoside, β-carotene, aluminium chloride, potassium bromide, potassium persulfate, sodium bicarbonate, sodium borate, sodium sulphate, sodium sulphite, cetrimonium bromide, citric acid, malic acid, and PMP (1-phenyl-3-methyl-5-pyrazolone) were acquired from Merck (Madrid, Spain). Pure compounds for the identification of monosaccharides, enzymes and electrolytes for in vitro digestion were also purchased from Merck. Bacterial strains *Lactobacillus casei* CECT 475 and *Lactococcus lactis* subsp. *lactis* CECT 185, as well as their respective culture media, were obtained from the Spanish Type Culture Collection (CECT) and PanReac, respectively.

### 2.2. Persimmon By-Product

Random by-product batches of persimmon (*Diospyros kaki* var. Rojo Brillante) from juice processing were received from Mitra Sol Technologies (Elche, Spain). By-product consisted of fresh pulp (70% moisture content) from the persimmon juice industry [9]. The by-products were tested for microbial contamination and deemed safe for use. The by-product was stored at −20 °C until needed for further experimentation.

### 2.3. Dietary Polysaccharides Purification

For the preparation of NADES, a mixture of citric acid: malic acid: water in a molar ratio of 1:1:10 was used [8]. The eutectic mixture was heated at 50 °C for 30 min with magnetic stirring until a clear and transparent liquid was obtained. To prepare the samples, 200 g of fresh persimmon pulp by-product (PPBP) was weighed and mixed with the NADES in a 1:5 ratio (solid to liquid) to 1 L as the final volume. The mixture was then sonicated for 15 min at 40 Hz amplitude. After treatment, the samples were centrifuged (10 min at 6370× *g*) to separate the processed fibre (pellet) and recover the eutectic extract (supernatant), containing free eutectic phytochemicals. The resulting polysaccharide pellets were washed, filtered and dried to obtain the persimmon pulp dietary fibre (PPDF). Both the PPDF and the eutectic extract were kept refrigerated (4 °C) for further analysis. The efficiency of the extraction was calculated per sample, based on the weight of the sample used for extraction. 

### 2.4. Extraction of Free and Bound Phytochemicals

Free phytochemicals are defined as the extractable fraction using traditional techniques. To extract free compounds from PPBP, 500 mg of dried and crushed (0.5 mm) sample was weighed in triplicate and mixed with 10 mL of 50% acetone. The mixture was stirred (2 h at 120 rpm) and centrifuged for 15 min at 2000× *g* at room temperature. The leftover pellet was removed, and the remaining liquid was frozen for later analysis. The free-conventional compounds were compared to the free-eutectic extract obtained during the PPDF purification process. To assess the bioactive potential of the by-products before and after treatment, fibre-bound phytochemicals were also studied. Dried samples of 500 mg (*n* = 3) were mixed with 10 mL of 50% acetone and then subjected to alkaline/acidic hydrolysis with 5 M NaOH and 5 M HCl, as previously described [10]. Centrifugation was performed for 15 min at 2000× *g* and the resulting liquid was snap frozen for later analysis. The efficiency of the extraction was calculated per sample, based on the weight of the sample used for extraction.

### 2.5. Determination of Phytochemicals

#### 2.5.1. Total Phenolic, Flavonoid, Carotenoid, and Tannin Content

Total phenolic content (TPC) [16], total flavonoid content (TFC) [17], and total carotenoid content (TCC) [18] were determined in the free-conventional, free-eutectic and bound phytochemical fractions from PPBP and PPDF after eutectic extraction. More information can be found in Supplementary Materials and Methods. Results were expressed as mg of gallic acid equivalents per g (mg GAE/g), mg of quercetin equivalents per g (mg QE/g), and mg of β-carotene equivalents per g (mg βCE/g), respectively. The absorbance of samples was determined by a microplate reader (Cytation™ 3 Cell Imaging Multi-Mode, BioTek, Winooski, VT, USA). In addition, the total tannin content (TTC) was also determined in samples using a method described in [19], and the results were expressed in mg of cyanidin-3 glucoside equivalents per g (mg C3GE/g) of sample. Phytochemicals in the eutectic extract content were expressed on a per-sample basis, which was calculated based on the weight of the sample used for the extraction. 

#### 2.5.2. Antioxidant Activity 

To assess the antioxidant activity of samples, DPPH^•^ and ABTS^•+^ assays were used for the extractable fractions, eutectic extract, and bound phytochemical fractions from PPBP and PPDF [20,21]. Different concentrations of Trolox (6-hydroxy-2,5,7,8-tetramethylchroman-2-carboxylic acid) ranging from 0 to 0.8 mM and 0 to 0.4 mM were used for quantification of the antioxidant activity in DPPH^•^ and ABTS^•+^, respectively. A microplate reader was used to determine the absorbance of samples at 515 nm for DPPH^•^ and 734 nm for ABTS^•+^. The results of both assays were expressed in mg of Trolox equivalent per sample basis, which was calculated based on the weight of the sample used for the extraction (mg TE/g). More information can be found in Supplementary Materials and Methods.

### 2.6. Polysaccharide Characterization

#### 2.6.1. Dietary Fibre Determination

For the determination of dietary fibre, differentiation was made between total dietary fibre (TDF), acid detergent fibre (ADF), which indicates the amount of cellulose and lignin, and neutral detergent fibre (NDF), which in addition determines the amount of hemicellulose present in the sample and has been reported to be fermentable by the gut microbiota [22,23,24]. ADF and NDF were used as indicators of structural changes in persimmon dietary fibre after US-NADES treatment. More information can be found in Supplementary Materials and Methods.

#### 2.6.2. Determination of the Monosaccharide Profile by Liquid Chromatography and Mass Spectrometry (LC-MS/MS)

Monosaccharide composition also provides information about the structure of the different polymeric fractions (following hydrolysis). For this reason, polysaccharides were hydrolysed and derivatised using PMP prior to LC-MS/MS analysis. PPBP and PPDF fractions (50 mg, in triplicate) were mixed with 5 mL of 2 M HCl at 90 °C for 3 h. The samples were cooled down to room temperature and centrifuged for 10 min (5300× *g*). The monosaccharide hydrolysate was stored at 4 °C prior to PMP derivatisation. Monosaccharide hydrolysate (100 μL), 0.5 M PMP methanolic solution (100 μL), and ammonia solution (100 μL) were mixed, heated at 70 °C for 30 min and then allowed to cool. Subsequently, 1 mL of 10% glacial acetic acid solution was added to stop the reaction. Then, the mixture was washed three times with 1 mL of pure chloroform and shaken for 1 min. The mixture was centrifuged for 10 min (5300× *g*). The upper aqueous phase was filtered through a 0.45 μm membrane for LC-MS/MS analysis.

Monosaccharide identification was performed by the optimisation of multiple reaction monitoring (MRM) conditions of authentic standards (Table S1) in an LC-MS/MS Shimadzu system, CBM-40A, with an SPD-M30A detector coupled to a triple quadrupole mass spectrometer analyser, equipped with an ESI interface and column oven CTO-20 AC type. Separation was performed on a column type Poroshell 120 SB-C18 2.7 µm (4.6 × 150 mm); the volume of injection was 1 μL. The mobile phase consisted of 10 mM of aqueous ammonium acetate solution (solvent A) and acetonitrile (solvent B). Chromatographic separation was achieved with the following gradient: 0–45 min, 20–30% B; 45–55 min, 30–20% B. The column was maintained at 30 °C.

#### 2.6.3. Polysaccharide Structure and Morphology Analysis

The PPBP and PPDF samples were subjected to Fourier-transformed infrared (FTIR) spectroscopy analysis by the potassium bromide (KBr) disc method. Samples were evenly mixed with dry KBr. Then, the sample, in an appropriate amount, was poured into a clean tableting mould. The infrared spectra were performed using a PerkinElmer Spectrum 3 FT-IR/NIR/FIR spectrometer over a frequency range between 4000 cm^−1^ and 500 cm^−1^. Pure KBr tablets were used as a reference background, and the spectra of samples were recorded as the average of 16 scans at 4 cm^−1^ resolution. 

The PPBP and PPDF samples were analysed with a field emission scanning electron microscope (FESEM) (Sigma 300 VP model, Carl Zeiss Microscopy GmbH, Oberkochen, Germany) at 15 kV and a magnification of 50 to 250× without coating.

#### 2.6.4. Polysaccharide Functional (Physical) Properties

To elucidate the techno-functional properties of PPBP and PPDF, a series of experiments were evaluated, such as wettability, specific volume, and water trapping capacity, as described previously [25]. Swelling and water holding capacity were determined according to [26], while oil holding capacity was determined according to [27]. In addition, physio-functional properties such as bile holding capacity and bioavailability analysis of monosaccharides after the in vitro digestion process [10,28] and fermentability indexes with probiotic strains of *L. casei* and *L. lactis* were gravimetrically assessed, as well as a recording of bacterial proliferation [29].

### 2.7. Beverage Formulation 

The beverages were prepared to mimic those available on the market; they consisted of smoothies with a total fibre concentration below 1% (*w*/*v*). The production of the beverages was performed at a pilot-scale facility. Three types of previously developed commercial beverages were prepared: isotonic, energy, and dairy-based using fruits and natural extracts as ingredients. To develop beverages considered a “Source of Fibre” [30], the addition of dietary fibre was carried out following the European Parliament and Council Regulation No. 1924/3006 regarding nutrition and health claims on food. For each new beverage, 3% (*w*/*v*) of PPDF was incorporated.

#### Sensory Evaluation

Thirty-seven panellists, ages 18–30, were recruited for this study. Sensory evaluation was carried out for the designed beverages. The tests were carried out at a controlled temperature (22 °C). For the assessment of the sensory profile, a 2-alternative forced choice (2-AFC) test [31], was performed. Subjects were instructed to taste each sample, indicate if they had identified any differences between the samples, and describe the nature of the differences in the measured characteristics. Furthermore, the panellists were asked to indicate their direction of preference. The following attributes were assessed: aroma, flavour, sweetness, astringency, acidity, and mouthfeel. The study was conducted in three sessions, each dedicated to a type of dietary fibre-added beverage (isotonic, energy or dairy-based) and its respective control (beverage without PPDF fibre). A 5-point hedonic scale was used for the evaluation, with 1 being the most pleasant and 5 being the most unpleasant. Sample order was randomised to limit sample order bias.

### 2.8. Statistical Analysis

GraphPad Prism 8.0.2 software (GraphPad Software, Inc., San Diego, CA, USA) was used for statistical analysis of the data, including analysis of variance (ANOVA), *t*-test, Mann–Whitney, Kruskal–Wallis, and Tukey post hoc tests, where applicable. All assays were performed in triplicate, and the data were reported as the mean ± standard deviation (SD). Statistical significance was set at *p* < 0.05.

## 3. Results

### 3.1. NADES Extraction and Extracts Composition

**Figure 1:**
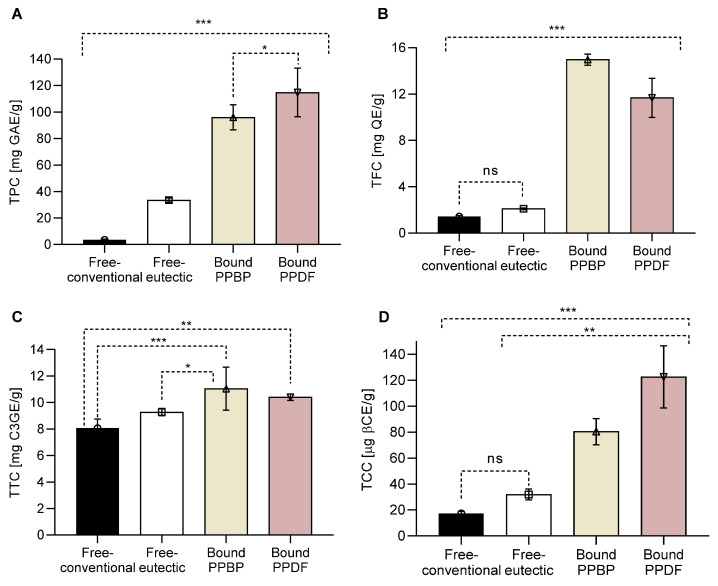
Phytochemical content of persimmon pulp by-product (PPBP) and persimmon pulp dietary fibre (PPDF) after ultrasonic treatment (US) in a natural deep eutectic solvent (NADES). (**A**) Total phenolic content (TPC), (**B**) total flavonoid content (TFC), (**C**) total tannin content (TTC), and (**D**) total carotenoid content (TCC) in the free-conventional, free-eutectic, and in bound PPBP and bound PPDF fractions. Overall, bound PPDF fraction showed the highest bioactive content (*** *p <* 0.001, ** *p <* 0.01, * *p <* 0.05, ns *p >* 0.05, one-way ANOVA with Tukey’s post hoc test). Values are expressed as mean (*n* = 3) ± standard deviation (SD).

The NADES treatment applied to persimmon by-products from juice production affected the structure of the pulp waste fibre-rich (polysaccharides) fraction, and consequently their phytochemical composition (Figure 1). 

A nine-fold increase in total phenolic content (TPC) was measured in the free-eutectic fraction in comparison with the free-conventional fraction (Figure 1A). The bound TPC in the PPDF fraction increased in comparison with the untreated bonded fraction in PPBP (*p <* 0.05), which had the highest TPC. The eutectic treatment has not affected the TFC amount (Figure 1B). Significant differences in TFC content were measured between treated and untreated fractions (*p <* 0.001), with the highest TFC in the bound PPBP fraction (before treatment). The bonded PPDP fraction had a nine-fold increase in the TFC over the free-conventional fraction.

An increase in TTC (Figure 1C) was measured in bound fractions (*p <* 0.001, PPBP vs. free-conventional and *p <* 0.01 PPDF vs. free-conventional). An increase in TCCs was detected in the bound samples, with a five-fold increase over the free-eutectic fraction (Figure 1D). Overall, bonded TCC in PPDF was significantly higher than in PPBP (*p <* 0.001).

### 3.2. Antioxidant Activity

**Figure 2:**
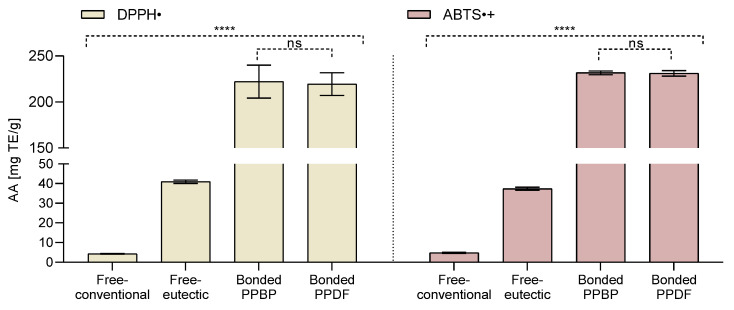
Antioxidant activity (AA) in the free-conventional, free-eutectic, and in bound persimmon pulp by-product (PPBP) and bound persimmon pulp dietary fibre (PPDF) fractions, measured by DPPH^•^ and ABTS^•+^ assays. The AA was expressed as milligram Trolox equivalent per gram fraction analysed (mg TE/g), where (**** *p <* 0.0001, ns *p >* 0.05, one-way ANOVA with Tukey’s post hoc test). Values are expressed as mean (*n* = 3) ± standard deviation (SD).

The antioxidant activity of extracted fractions was estimated using ABTS^•+^ and DPPH^•^ radical scavenging assays (see Figure 2). The bound compounds in the PPDF and PPBP fractions exhibited higher (50-fold) antioxidant activity than the free-available and free-eutectic fractions (*p <* 0.0001), for both assays. A ten-fold increase (for DPPH^•^ assay) and an eight-fold increase (for ABTS^•+^ assay) in the antioxidant activity for the free-eutectic fraction over the free-conventional fraction was observed (*p* < 0.0001). 

### 3.3. Polysaccharide Fractions

**Figure 3:**
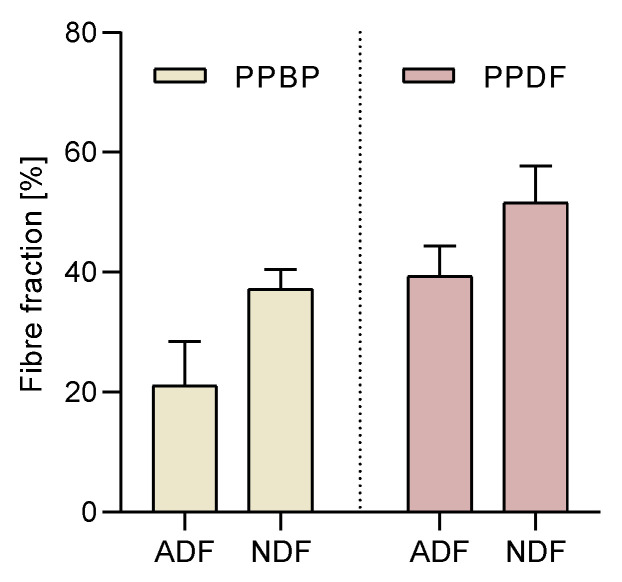
Acidic detergent fibre (ADF) and neutral detergent fibre (NDF) fractions in persimmon pulp by-product (PPBP) and persimmon pulp dietary fibre (PPDF) samples. An increment of total dietary fibre was found between PPBP and PPDF (*p* < 0.05); however, individual increases in ADF or NDF were not significant (*p* > 0.05; two-way ANOVA with Tukey’s post hoc test). Values are expressed as mean (*n* = 3) ± standard deviation (SD).

The effects of processing on the polysaccharide structure (NDF and ADF fractions) in PPBP samples were in a 2:1 ratio, while in PPDF samples they were in a 1:1 ratio. (Figure 3). No significant differences were found between the NDF and ADF content before and after treatment; however, a significant increase in the total dietary fibre after treatment was found (*p* < 0.05). 

### 3.4. Monosaccharide Identification

**Figure 4:**
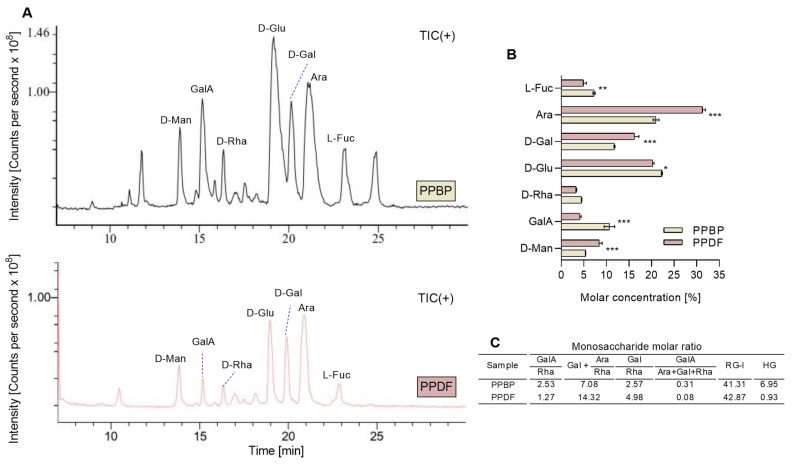
The monosaccharide profile chromatograms of persimmon pulp by-product (PPBP) and persimmon pulp dietary fibre (PPDF) (**A**). The molar concentration of monosaccharides in PPBP and PPDF samples (**B**). The molar ratios of monosaccharides found in PPBP and PPDF (**C**). Where the homogalacturonan, HG = GalA − Rha, and the rhamnogalacturonan, RG-I = 2 × Rha + Ara + Gal. A higher molar concentration of Ara, D-Gal, and D-Man was found in PPDF than in PPBP (*** *p* < 0.001, ** *p* < 0.01, * *p* < 0.05; Student’s *t*-test). Values are expressed as mean (*n* = 3) ± standard deviation (SD).

MRM analysis of the PMP-derivatised products of PPBP and PPDF samples following the acid hydrolysis showed seven ion peaks (Figure 4A) corresponding to the same pattern of monosaccharides. The fibre monosaccharide spectrum profile changed before and after the treatment. The main monosaccharides identified were glucose (Glu) at 19.6 min, arabinose (Ara) at 21.4 min, galactose (Gal) at 20.2 min, galacturonic acid (GalA) at 15.3 min, fucose (Fuc) at 23.1 min, mannose (Man) at 13.9 min, and rhamnose (Rha) at 16.3 min. Figure 4B displays the molar concentration of monosaccharides, with Ara, Glu and Gal being the most predominant monosaccharides before and after treatment. Ara and Gal increased after treatment, while GalA decreased. Molar ratios in Figure 4C showed the rhamnogalacturonan (RG-I) as predominating, and the value of (Gal + Ara)/Rha indicates an extensive branching of RG-I segments. The low value of GalA/(Ara + Gal + Rha) implies a limited linear chain, and the value of Gal/Rha suggests that the RG-I regions of PPDF and PPBP contain long galactan sidechains.

### 3.5. Polysaccharide Structure 

**Figure 5:**
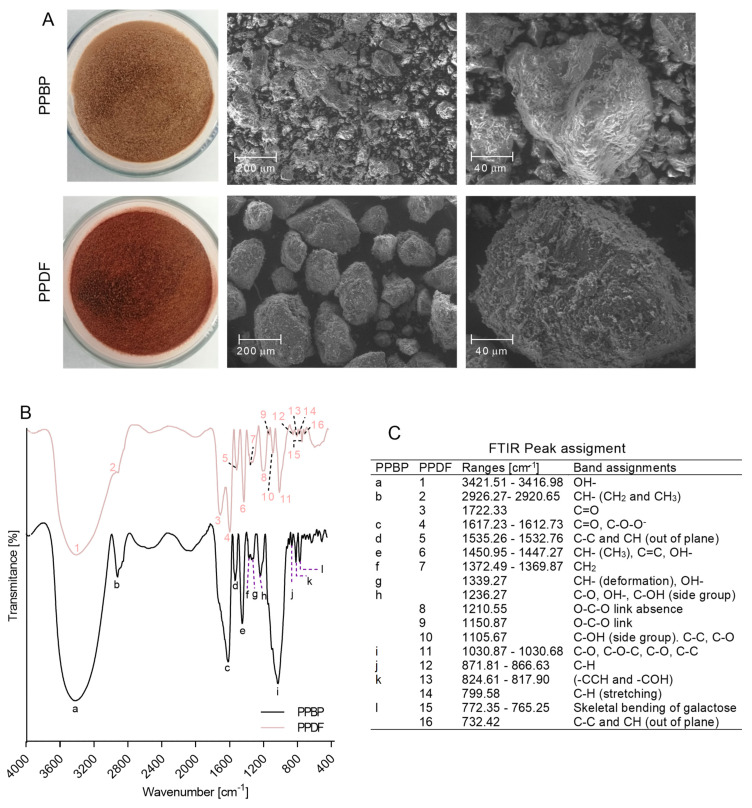
(**A**) Field emission scanning electronic microscope (FESEM) micrographs of persimmon pulp by-product (PPBP) and persimmon pulp dietary fibre (PPDF) samples. (**B**) Fourier-transformed infrared (FTIR) spectra of PPBP and PPDF samples. (**C**) List of peaks from PPBP and PPDF samples FTIR spectra (cm^−1^) and their assignments.

The FESEM analysis showed morphologic changes in the persimmon polysaccharide matrix and physical aspects after treatment with NADES (Figure 5A). The PPBP sample consisted of granular aggregated structures composed of shaped medium-sized granules, while the PPDF sample consisted mainly of aggregated globular particles with a cleaner surface than PPBP. 

The FTIR (Figure 5B) displayed functional groups that lead to the aggregation of polysaccharides, with the bonds of PPBD and PPDF being similar in the single bond stretch region, with a wide, strong peak in 3416.98–3421.51 cm^−1^ that corresponds to the hydroxyl groups of cellulose, hemicellulose, and tannic acid [32,33], as well as a peak in 2926.27–2920.65 cm^−1^ (corresponding to the methylene groups of saccharides such as pectin) [32,34].

In the double bond region, a sharp peak was observed in 1617.23–1612.73 cm^−1^ bands, indicating the presence of carbonyl groups of aromatic compounds such as aldehydes and ketones, as well as free carboxylic groups of poly-GalA and gallo-tannins [34,35,36,37]. A weak peak was also observed in both samples in the 1535.26–1532.76 cm^−1^ region (corresponding to methyl alkene groups of C–C and CH out-of-plane bonds of phenolic compounds such as proanthocyanidins) [35,38]. 

The 1450.95–1447.27 cm^−1^ bands in both samples indicated the presence of asymmetric stretching modes of vibration of methyl esters reported in pectin, the benzene ring of phenols and the flavan units, as well as the plane bending vibrations of hydroxyl groups in phenols [35,36,39]. The weak peak in the 1372.49–1369.87 cm^−1^ region indicates the presence of methylene groups in saccharides [36,37], and the peak in the 1030.87–1030.68 cm^−1^ region indicates the glycosidic groups in phenolics, pectin, Ara-based glucans and the glycosidic bonds in cellulose, glucans, and pentoses [35,36,37,40,41]. Both samples also showed a similar spectrum in the regions from 871.81–866.63 cm^−1^ (corresponding to Man-containing polysaccharides) [36], the 824.61–817.90 cm^−1^ region (indicating bending at the C6 position of Gal unit) [32,42], and the 772.35–765.25 cm^−1^ peak (as skeletal bending of Gal) [42].

The spectra for the PPBP sample showed peaks in the regions of 1339.27 cm^−1^ (corresponding to CH deformation in pectin, cellulose, Fuc and poly- GalA, as well as hydroxyl groups of phenolics) [36,37,43], and 1236.27 cm^−1^ region (indicating carbonyl groups of pectin and/or rhamnogalacturonan, Glu and hydroxyl and the C–OH side group of phenols) [35,36,37,43]. After treatment, in PPDF sample spectra, these peaks disappeared, and new peaks were observed in the regions of 1722.33 cm^−1^ (corresponding to ester carbonyl groups of polysaccharides and hemicellulose or lignin). Additionally, characteristic peaks were also observed for polyphenols such as gallic acid, tannic acid, quercetin and rutin [33,36,39], peaks at 1210.55–1150.87 cm^−1^ (corresponding to glycosidic bonds of GalA, Man-containing hemicellulose, cellulose, pectin, Gal and arabinoxylan) [36,37,40], at 1150.87 cm^−1^ (for C–OH side groups of glycosidic linkages between sugar units, phenolics, as well as for the glycosidic bonds of C–C in phenolics and for C–O in saccharides) [32,35,39], at 799.58 cm^−1^ (indicating C–H stretching of the benzene ring of phenols) [34], and peak at 732.42 cm^−1^ (for methyl alkene C–C and CH out-of-plane bonds of phenolic type proanthocianidins) [38].

### 3.6. Functional Properties of Fibre

**Figure 6:**
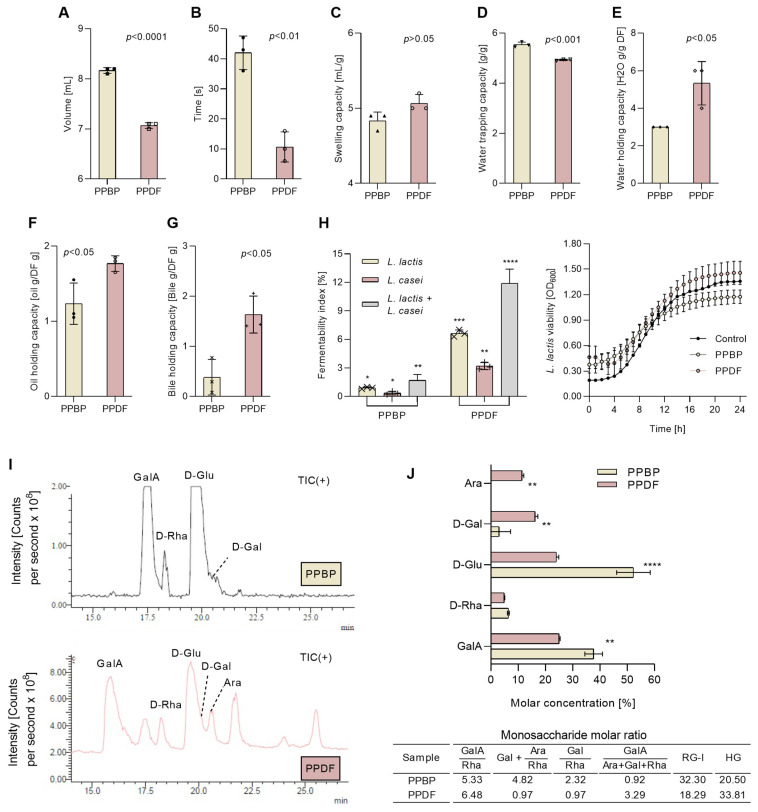
The physical and physio-functional properties of persimmon pulp by-product (PPBP) and persimmon pulp dietary fibre after treatment (PPDF). The physical properties, the wettability (**A**), the specific volume (**B**); the techno-functional properties, the swelling capacity (**C**), the water trapping capacity (**D**), the water holding capacity (**E**), and the oil holding capacity (**F**); and physio-functional properties, the bile holding capacity (**G**), the fermentability index (**H**), and the digestibility (**I**), and the molar concentration and ratio of digested monosaccharides (**J**). Following the PPBP fibre fraction treatment, the PPDF samples showed higher water holding, swelling, oil holding capacity, bile holding capacities (**** *p <* 0.0001, *** *p <* 0.001, ** *p <* 0.01, * *p <* 0.05, ns *p >* 0.05, Student’s *t*-test), and fermentability index (**** *p <* 0.0001, *** *p <* 0.001, ** *p <* 0.01, * *p <* 0.05, two-way ANOVA with Tukey’s post hoc test) than PPBP. Values are expressed as mean (*n* = 3) ± standard deviation (SD).

The physical properties of the fibre fraction obtained after treatment changed significantly, as did the physio-functional properties, in comparison with the untreated persimmon fibre (Figure 6). Before any treatment, the fibre fraction occupied a higher volume (*p* < 0.001), had a higher wettability (*p* < 0.01), and exhibited a greater water trapping capacity (*p* < 0.001) than treated fibre (PPDF sample) (Figure 6A,B,D). The treatment improved the swelling capacity (*p* < 0.05) (Figure 6C), the water holding capacity (*p* < 0.05) (Figure 6E), the oil holding capacity (*p* < 0.05) (Figure 6F), and the bile holding capacity (*p* < 0.05) (Figure 6G). The treatment also enhanced the fibre fermentability (Figure 6H), as *L. lactis* showed a higher preference for PPDF and fermented up to 10% of the TDF. Moreover, the optical densities at 600 nm (OD600) values for PPDF, PPBP samples and the culture medium inoculated with *L. lactis* followed a similar bacterial growth pattern, increasing during the first 16 h and stabilising afterwards (16–24 h). The combination of *L. lactis* and *L. casei* showed a synergistic effect and increased fermentability rates up to 15% for the PPDF sample (Figure 6H). 

In the case of sample digestibility before the treatment, PPBP (Figure 6I) the GalA elution time was observed displaced at 17.5 min and lower amounts of monosaccharides were released, corresponding to GalA, Glu, Rha and Gal. Following the digestion of the treated sample (PPDF), a five-fold increase in GalA, a one-fold increase in Glu, and a two-fold increase in Gal and Ara were found to be released. The molar ratio of digested monosaccharides showed that the modest fractions GalA from the homogalacturonan (HG) domain, the short branches containing galactans from the RG-I domain, and the hemicellulose branches are hydrolysable and are released after digestion.

### 3.7. Sensory Profile of the Beverages

**Figure 7:**
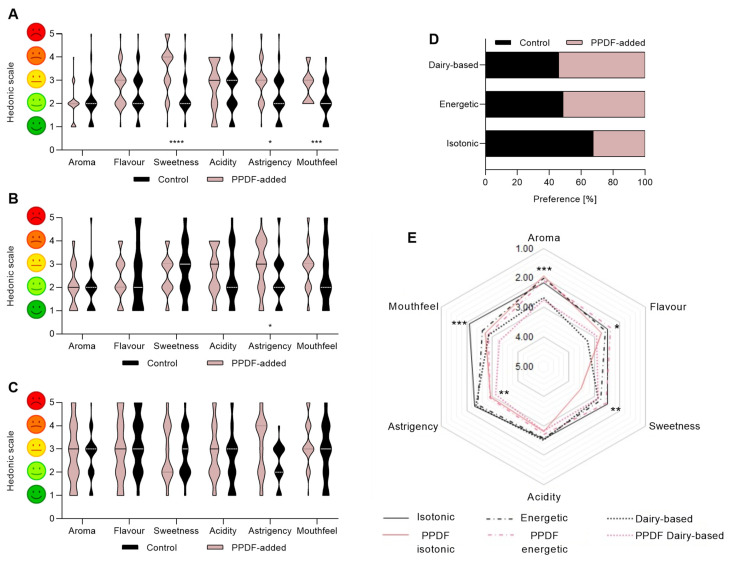
Sensorial analysis of beverage formulations for isotonic drink (**A**), energy drink (**B**), and dairy-based drink (**C**) with PPDF-added vs. control. The PPDF-added dairy-based formula showed similar appreciation by panellists when compared with control (non-PPDF-added beverage), while panellists showed less appreciation for the PPDF-added isotonic formula in comparison with the control (where, **** *p <* 0.0001, *** *p <* 0.001, * *p <* 0.05, Student’s *t*-test with Mann–Whitney post hoc test). The preference of consumption for each beverage in comparison with its control (**D**). The PPDF-added dairy-based beverage showed more than 50% of preference in comparison with the control beverage. The global comparison of attributes of all samples (**E**). Overall, the smell, taste and mouthfeel attributes were more appreciated in PPDF-added formulas than in the control samples (with *** *p <* 0.001, ** *p <* 0.01, * *p <* 0.05, one-way ANOVA with Kruskal–Wallis post hoc test). The values are expressed as mean (*n* = 3) ± standard deviation (SD).

For the 2-AFC sensory attributes evaluation of the isotonic beverage, the panellists found no significant preferences between beverages for aroma, flavour, and acidity (in comparison with the control, the non-PPDF-added beverage) (Figure 7A). However, for the astringency, the mouth sensation, and the sweetness, panellists followed a significant pattern of preference, with a two-unit difference between the beverages (the widest gap between samples). In terms of forced preference between the beverage with fibre and the control sample, 68% of the panellists chose the sample without fibre (Figure 7D). For the energy drink (Figure 7B), the panellists found a difference in astringency (*p <* 0.0001) in comparison with the control beverage. A high variability in the panellists’ responses was observed when evaluating the sweetness of the beverage. No statistical preference was found between the beverages when tested for the other beverage attributes. In the forced preference, 51% of the panellists chose the dairy beverage with fibre (Figure 7D).

According to the panellists, for the dairy-based beverage (Figure 7C), there was no statistical preference between the beverage with or without fibre for any of the attributes measured. However, a variability of up to 97.55% and, respectively, 82.94% (*p <* 0.0001) was detected among panellists evaluating the astringency and acidity properties. In the forced preference, 54% of the panellists chose the dairy-based beverage with fibre (Figure 7D) over the control beverage. Overall analysis (Figure 7E) led to discrimination among beverages and found significant differences in all properties except for acidity (*p <* 0.0001). For qualities such as aroma or flavour, the best scores were for the beverages with fibre; the aroma for the isotonic drink (*p <* 0.0001) and the flavour for the energy drink (*p <* 0.0001). Regarding the sweetness and the mouthfeel, the isotonic drink without fibre was preferred. Finally, for the astringency, a similar forced choice was reported among most of the beverages.

## 4. Discussion

To our knowledge, this is the first report on the characterization and use of processed PPBP for beverage reformulation. The use of ultrasonic treatment (US) in a natural deep eutectic solvent (NADES) proved to be a very efficient approach to extracting and treating high-fibre by-products from the persimmon juice industry, as it promoted the extraction and release of significant amounts of fibre-bound bioactive phytochemicals. 

The US treatment could therefore emerge as a feasible processing technology to be used in both industry and research applications, being more efficient than existing traditional extraction methods. This is because the total phenolic, flavonoid, carotenoid and tannin contents were significantly increased in all the fractions after the treatment. The concentrations of polyphenols extracted using this technology were found to be much higher than those measured in other studies on fibre-rich persimmon samples [44]. The total flavonoid concentration was comparable to that of other fruits and vegetables [45]. The amount of carotenoids extracted from persimmon using the US-NADES was also found to be much higher than the one recorded through conventional extraction techniques [46]. However, the amount of tannin extracted with this treatment was found to be similar to that previously measured in this fruit [35].

We have previously evaluated the polyphenolic and carotenoid profiles in free and bound fractions from PPDF [9,10,14,47], where gallic acid was the main released compound from persimmon fibre after digestion while β-cryptoxanthin was the main carotenoid found in persimmon. In this study, the free and bound phenolics content in the fibre-rich fractions contributed to its antioxidant activity, as the presence of GalA as well as the amount of unmethylated carboxylic groups supplied hydrogen ions (H^+^), which combined to form a more stable radical with the ABTS^•+^ and DPPH^•^ radicals [48,49,50]. The results showed that the bound phytochemicals from persimmon fractions after US-NADES treatment displayed higher antioxidant activity than the untreated fraction. This suggests that these bioactive compounds (polyphenols, carotenoids, and monosaccharides), once released throughout the gastrointestinal tract during the digestion and fermentation processes, could provide beneficial health effects that may be relevant as functional ingredients to be used in bakery products, snack bars, and beverages, increasing their nutritional value and shelf life. These antioxidant activity results were correlated with TPC, TFC, TCC, and TTC contents and are in alignment with results from other research studies on different persimmon varieties [44] and with previous functional properties found in persimmon-bonded compounds such as antibacterial and intestinal barrier-function promotion effects after in vitro digestion [10,14]; however, it would be necessary to conduct cell-based studies to provide a more comprehensive understanding of their antioxidant properties. 

The decrease in hemicellulose content after the treatment of persimmon fractions corresponds to polysaccharide hydrolysis, resulting in the loss of monomeric and oligomeric branched fractions under acidic conditions. The differences in composition between TDF, ADF, and NDF fractions reflect the pectic domains linked to the polysaccharide matrix, which increased after ultrasound-NADES treatment. The ADF and NDF composition data correlate with monosaccharide content in Glu concentration. The amounts of NDF and ADF were much higher than those measured from the fibre-rich by-products of other fruits such as the peel of mandarins [24]. 

Pectic polysaccharides have been described in persimmon, with the HG ratio being higher in the water-soluble fractions. The HG and RG-I are covalently bound in the fruit before processing. The presence of a high amount of Ara suggests the existence of (glucurono)arabinoxylans together with the prevalence of the RG-I. These results suggest that the main components of the PPBP and PPDF fractions are cellulose, hemicellulose, and pectin types. The high percent of pectic domains implies a vast heterogeneity of the PPBP fraction. The processing led to a decrease in the insoluble fibre delivering a lower percentage of hemicellulose. The composition of both PPBP and PPDF fractions showed an abundance of Man to hemicellulose. Hemicellulose and RG-I are fibres partially fermented by the microbiome, therefore contributing to the formation of the faecal bulk.

The FTIR spectra confirmed the data for monosaccharide identification for both PPBP and PPDF fractions, suggesting that US treatment of PPBP in NADES led to the loss of functional groups related to polysaccharides and tannins. Moreover, new functional groups were observed in PPDF, corresponding to monosaccharides and available polyphenols. During persimmon ripening, the loss of astringency is driven by tannins insolubilisation; these non-extractable polyphenols are covalently linked to the monosaccharide terminals within the polysaccharide branches.

The fibre fraction following the treatment presented a lower specific volume and a lower wettability time, due to structural factors such as the presence of the bound phytochemicals and their functional groups. For swelling capacity and wettability, this study’s results are similar to those reported for fractions obtained following conventional processing methods, while only the water retention capacity of the treated persimmon fibre was within those ranges. Treated and untreated persimmon fibre-rich fractions released small amounts of pectic monosaccharides that belonged to the HG domain, with the Glu monomers resulting from the hemicellulose and cellulose fractions and the Ara from the branched RG-I domain and arabinogalactans. Superior technological properties of the persimmon by-products were observed in previous studies; however, these involved in their production the use of more expensive and less green technologies such as lyophilization and organic solvents such as acetone and ethanol. The fermentability indexes for PPDF showed that probiotic strains are able to utilise the polysaccharides in their composition more efficiently than in the case of untreated fibres, suggesting that the treatment could be used to increase the fermentability of the persimmon by-product fibres.

Astringency is an important aspect of a food’s sensorial profile. The panellists were forced to perceive astringency attributes coming from persimmon fibre in three reformulated beverages (isotonic, energy, and dairy-based), and this has been found in the taste of the two novel beverages. The results from the sensorial testing of the reformulated beverages showed that there was no significant difference between the control beverage and the beverage with fibre, suggesting that dietary fibre could be easily added to beverages and be accepted by consumers. 

## 5. Conclusions

The US-NADES fibre processing improved polysaccharide qualities, resulting in a greater extractability of phytochemicals, antioxidant capacity, and an improvement in technological functionality and fermentability, in addition to improving the sensorial properties once used for beverage reformulations. The persimmon fibre-rich fraction by-products from the juice industry were a rich source of functional monosaccharides such as GalA, Rha, and Ara, therefore being a sustainable source of dietary fibre and a source of bioactive phytochemicals with antioxidant properties to be used for food formulation and the development of functional foods such as prebiotics. 

## Data Availability

Not applicable.

## Supplementary Materials

The following supporting information can be downloaded at: https://www.mdpi.com/article/10.3390/antiox12051085/s1, S1: Supplemental Materials and Methods. Table S1: Annotation and detection conditions for monosaccharide analysis.

## References

[1] Sharma S.K., Bansal S., Mangal M., Dixit A.K., Gupta R.K., Mangal A.K. (2016). Utilization of Food Processing By-products as Dietary, Functional, and Novel Fiber: A Review. Crit. Rev. Food Sci. Nutr..

[2] FAO (2014). Inputs for FAO’s Contribution to the 2014 ECOSOC. Presented at: Integration Segment of the Economic and Social Council Focused on “Sustainable Urbanization”. https://www.un.org/en/ecosoc/integration/pdf/foodandagricultureorganization.pdf.

[3] Baiano A. (2014). Recovery of biomolecules from food wastes—A review. Molecules.

[4] Torres-León C., Ramírez-Guzman N., Londoño-Hernandez L., Martinez-Medina G.A., Díaz-Herrera R., Navarro-Macias V., Alvarez-Pérez O.B., Picazo B., Villarreal-Vázquez M., Ascacio-Valdes J. (2018). Food Waste and Byproducts: An Opportunity to Minimize Malnutrition and Hunger in Developing Countries. Front. Sustain. Food Syst..

[5] Carlson J.L., Erickson J.M., Lloyd B.B., Slavin J.L. (2018). Health Effects and Sources of Prebiotic Dietary Fiber. Curr. Dev. Nutr..

[6] Hui C.Y., Lee K.C., Chang Y.P. (2022). Cellulase-Xylanase-Treated Guava Purée by-Products as Prebiotics Ingredients in Yogurt. Plant Foods Hum. Nutr..

[7] Dong W., Yang Z. (2022). Association of Dietary Fiber Intake With Myocardial Infarction and Stroke Events in US Adults: A Cross-Sectional Study of NHANES 2011-2018. Front. Nutr..

[8] Santana A.P.R., Mora-Vargas J.A., Guimarães T.G.S., Amaral C.D.B., Oliveira A., Gonzalez M.H. (2019). Sustainable synthesis of natural deep eutectic solvents (NADES) by different methods. J. Mol. Liq..

[9] Gea-Botella S., Agulló L., Martí N., Martínez-Madrid M.C., Lizama V., Martín-Bermudo F., Berná G., Saura D., Valero M. (2021). Carotenoids from persimmon juice processing. Food Res. Int..

[10] Salazar-Bermeo J., Moreno-Chamba B., Martínez-Madrid M.C., Saura D., Valero M., Martí N. (2021). Potential of Persimmon Dietary Fiber Obtained from Byproducts as Antioxidant, Prebiotic and Modulating Agent of the Intestinal Epithelial Barrier Function. Antioxidants.

[11] Santos A.D.D.C., Fonseca F.A., Dutra L.M., Santos M.F.C., Menezes L.R.A., Campos F.R., Nagata N., Ayub R., Barison A. (2018). H HR-MAS NMR-based metabolomics study of different persimmon cultivars (*Diospyros kaki*) during fruit development. Food Chem..

[12] González C.M., Hernando I., Moraga G. (2021). In Vitro and In Vivo Digestion of Persimmon and Derived Products: A Review. Foods.

[13] Akter S., Eun J.-B. (2009). Characterization of Insoluble Fibers Prepared from the Peel of Ripe Soft Persimmon (*Diospyros kaki* L. cv. Daebong). Food Sci. Biotechnol..

[14] Moreno-Chamba B., Salazar-Bermeo J., Martínez-Madrid M.C., Lizama V., Martín-Bermudo F., Berná G., Neacsu M., Saura D., Martí N., Valero M. (2022). Bound galloylated compounds in persimmon upcycled dietary fiber modulate microbial strains associated to human health after in vitro digestion. LWT.

[15] Asgar A., Yamauchi R., Kato K. (2003). Modification of pectin in Japanese persimmon fruit during the sun-drying process. Food Chem..

[16] Singleton V.L., Orthofer R., Lamuela-Raventós R.M. (1999). Analysis of total phenols and other oxidation substrates and antioxidants by means of folin-ciocalteu reagent. Methods in Enzymology.

[17] Pękal A., Pyrzynska K. (2014). Evaluation of Aluminium Complexation Reaction for Flavonoid Content Assay. Food Anal. Methods.

[18] Nagata M., Yamashita I. (1992). Simple method for simultaneous determination of chlorophyll and carotenoids in tomato fruit. Nippon. Shokuhin Kogyo Gakkaishi.

[19] Ribéreau-Gayon P., Glories Y., Maujean A., Dubourdieu D., Sons J.W. (2006). Phenolic compounds. Handbook of Enology: The Chemistry of Wine Stabilization and Treatments.

[20] Brand-Williams W., Cuvelier M.E., Berset C. (1995). Use of a free radical method to evaluate antioxidant activity. LWT-Food Sci. Technol..

[21] Re R., Pellegrini N., Proteggente A., Pannala A., Yang M., Rice-Evans C. (1999). Antioxidant activity applying an improved ABTS radical cation decolorization assay. Free Radic. Biol. Med..

[22] Van Soest P.J., Robertson J.B., Lewis B.A. (1991). Methods for dietary fiber, neutral detergent fiber, and nonstarch polysaccharides in relation to animal nutrition. J. Dairy Sci..

[23] Mertens D.R. (2002). Gravimetric determination of amylase-treated neutral detergent fiber in feeds with refluxing in beakers or crucibles: Collaborative study. J. AOAC Int..

[24] Martí N., Saura D., Fuentes E., Lizama V., García E., Mico-Ballestera M.J., Lorente J. (2011). Fiber from tangerine juice industry. Ind. Crops Prod..

[25] Hinestroza-Córdoba L.I., Duarte Serna S., Seguí L., Barrera C., Betoret N. (2020). Characterization of Powdered Lulo. Foods.

[26] Raghavendra S.N., Rastogi N.K., Raghavarao K.S.M.S., Tharanathan R.N. (2004). Dietary fiber from coconut residue: Effects of different treatments and particle size on the hydration properties. Eur. Food Res. Technol..

[27] Robertson J.A., de Monredon F.D., Dysseler P., Guillon F., Amado R., Thibault J.F. (2000). Hydration properties of dietary fibre and resistant starch: A European collaborative study. LWT-Food Sci. Technol..

[28] Minekus M., Alminger M., Alvito P., Ballance S., Bohn T., Bourlieu C., Carrière F., Boutrou R., Corredig M., Dupont D. (2014). A standardised static in vitro digestion method suitable for food—An international consensus. Food Funct..

[29] Zhang J., Chen H., Luo L., Zhou Z., Wang Y., Gao T., Yang L., Peng T., Wu M. (2021). Structures of fructan and galactan from Polygonatum cyrtonema and their utilization by probiotic bacteria. Carbohydr. Polym..

[30] Parlamento Europeo y del Consejo (2014). (UE) R. Reglamento (CE) No 1924/2006 Relativo a las Declaraciones Nutricionales y de Propiedades Saludables en los Alimentos. 1924/2006: Diario Oficial de la Unión Europea.

[31] Rousseau B., O’Mahony M. (1997). Sensory difference tests: Thurstonian and SSA predictions for vanilla flavored yogurts. J. Sens. Stud..

[32] Muñoz-Almagro N., Vendrell-Calatayud M., Méndez-Albiñana P., Moreno R., Cano M.P., Villamiel M. (2021). Extraction optimization and structural characterization of pectin from persimmon fruit (*Diospyros kaki* Thunb. var. *Rojo brillante*). Carbohydr. Polym..

[33] Patle T.K., Shrivas K., Kurrey R., Upadhyay S., Jangde R., Chauhan R. (2020). Phytochemical screening and determination of phenolics and flavonoids in *Dillenia pentagyna* using UV-vis and FTIR spectroscopy. Spectrochim. Acta Part A Mol. Biomol. Spectrosc..

[34] Liu S., Jia M., Chen J., Wan H., Dong R., Nie S., Xie M., Yu Q. (2019). Removal of bound polyphenols and its effect on antioxidant and prebiotics properties of carrot dietary fiber. Food Hydrocoll..

[35] Liu M., Wang J., Yang K., Qi Y., Zhang J., Fan M., Wei X. (2018). Optimization of ultrasonic-assisted extraction of antioxidant tannin from young astringent persimmon (*Diospyros kaki* L.) using response surface methodology. J. Food Process. Preserv..

[36] Liu X., Renard C.M.G.C., Bureau S., Le Bourvellec C. (2021). Revisiting the contribution of ATR-FTIR spectroscopy to characterize plant cell wall polysaccharides. Carbohydr. Polym..

[37] Ying D., Hlaing M.M., Lerisson J., Pitts K., Cheng L., Sanguansri L., Augustin M.A. (2017). Physical properties and FTIR analysis of rice-oat flour and maize-oat flour based extruded food products containing olive pomace. Food Res. Int..

[38] Wahyono T., Astuti D.A., Wiryawan I.K.G., Sugoro I., Jayanegara A. (2019). Fourier transform mid-infrared (FTIR) spectroscopy to identify tannin compounds in the panicle of sorghum mutant lines. IOP Conference Series: Materials Science and Engineering.

[39] Zhang Y., Li X., Gong L., Xing Z., Lou Z., Shan W., Xiong Y. (2019). Persimmon tannin/graphene oxide composites: Fabrication and superior adsorption of germanium ions in aqueous solution. J. Taiwan Inst. Chem. Eng..

[40] Jiang Y., Xu Y., Li F., Li D., Huang Q. (2020). Pectin extracted from persimmon peel: A physicochemical characterization and emulsifying properties evaluation. Food Hydrocoll..

[41] Xue Z., Chen Y., Jia Y., Wang Y., Lu Y., Chen H., Zhang M. (2019). Structure, thermal and rheological properties of different soluble dietary fiber fractions from mushroom *Lentinula edodes* (Berk.) Pegler residues. Food Hydrocoll..

[42] Shanura-Fernando I.P., Asanka-Sanjeewa K.K., Samarakoon K.W., Woo-Lee W., Kim H.S., Kim E.A., Gunasekara U.K.D.S.S., Abeytunga D.T.U., Nanayakkara C., de Silva E.D. (2017). FTIR characterization and antioxidant activity of water soluble crude polysaccharides of Sri Lankan marine algae. Algae.

[43] Ye H., Luo L., Wang J., Jiang K., Yue T., Yang H. (2022). Highly galloylated and A-type prodelphinidins and procyanidins in persimmon (*Diospyros kaki* L.) peel. Food Chem..

[44] Martínez-Las Heras R., Landines E.F., Heredia A., Castelló M.L., Andrés A. (2017). Influence of drying process and particle size of persimmon fibre on its physicochemical, antioxidant, hydration and emulsifying properties. J. Food Sci. Technol..

[45] Lin J.Y., Tang C.Y. (2007). Determination of total phenolic and flavonoid contents in selected fruits and vegetables, as well as their stimulatory effects on mouse splenocyte proliferation. Food Chem..

[46] Veberic R., Jurhar J., Mikulic-Petkovsek M., Stampar F., Schmitzer V. (2010). Comparative study of primary and secondary metabolites in 11 cultivars of persimmon fruit (*Diospyros kaki* L.). Food Chem..

[47] Gea-Botella S., Moreno-Chamba B., de la Casa L., Salazar-Bermeo J., Martí N., Martínez-Madrid M.C., Valero M., Saura D. (2021). Carotenoids from Persimmon (*Diospyros kaki* Thunb.) Byproducts Exert Photoprotective, Antioxidative and Microbial Anti-Adhesive Effects on HaCaT. Pharmaceutics.

[48] Yang L., Zhao Y., Huang J., Zhang H., Lin Q., Han L., Liu J., Wang J., Liu H. (2020). Insoluble dietary fiber from soy hulls regulates the gut microbiota in vitro and increases the abundance of bifidobacteriales and lactobacillales. J. Food Sci. Technol..

[49] Yuan Y., Li C., Zheng Q., Wu J., Zhu K., Shen X., Cao J. (2019). Effect of simulated gastrointestinal digestion in vitro on the antioxidant activity, molecular weight and microstructure of polysaccharides from a tropical sea cucumber (*Holothuria leucospilota*). Food Hydrocoll..

[50] Olawuyi I.F., Lee W.Y. (2021). Structural characterization, functional properties and antioxidant activities of polysaccharide extract obtained from okra leaves (*Abelmoschus esculentus*). Food Chem..

