# Supplementary material for: Green Technologies for Persimmon By-Products Revalorisation as Sustainable Sources of Dietary Fibre and Antioxidants for Functional Beverages Development
Source: Antioxidants (Basel). 2023 May 12;12(5):1085. doi: 10.3390/antiox12051085 (PMC10215573; doi:10.3390/antiox12051085)
Supplement: Supplementary file 1 [file antioxidants-12-01085-s001.zip › antioxidants-2358346-supplementary.pdf]

antioxidants

MDPI

Supplementary Materials: Green Technologies for Persimmon By-products Revalorisation as Sustainable Sources of Dietary Fibre and Antioxidants for Functional Beverages Development

Julio Salazar-Bermeo, Bryan Moreno-Chamba, Rosa Heredia-Hortigüela, Victoria Lizama, María Concepción Mar-tínez-Madrid, Domingo Saura, Manuel Valero, Madalina Neacsu, and Nuria Martí

S1. Materials and Methods

S1.1. Determination of Phytochemicals

S1.1.1. Total phenolic content (TPC)

For the TPC assay, the Folin-Ciocalteu's reagent method was used [1]. Briefly, a volume of each sample: free-conventional, free-eutectic, and bound phytochemicals from persimmon pulp by-product (PPBP) and persimmon pulp dietary fibre (PPDF), was diluted in 80% methanol. First, 50 μL of each sample was mixed with 50 μL of Folin-Ciocalteu's reagent and incubated for 8 min in darkness at room temperature. Then, 150 μL of 20% sodium carbonate solution were added and the mixture was further incubated 30 min in darkness at room temperature. The absorbance was read at 765 nm using a microplate reader. To evaluate the intrinsic absorption of samples, 50 μL of methanol was added instead of the Folin-Ciocalteu's reagent. The reagent blank was evaluated by the addition of 50 μL of pure water instead of standard compound or sample. The results were expressed on a per-sample basis, which was calculated based on the weight of the sample used for the extraction. Gallic acid was used as a standard to create a calibration curve, and results were expressed as milligrams of gallic acid equivalents per gram of sample (mg GAE/g).

S1.1.2. Total flavonoid content (TFC)

The TFC assay used the aluminium chloride colorimetric method [2]. Briefly, a volume of each sample: free-conventional, free-eutectic, and bound phytochemicals from PPBP and PPDF, was diluted in 80% methanol; then, samples were mixed with 2% of AlCl3:6H2O methanolic solution in a 1:1 ratio in a 96-well plate (300 μL as final volume). After incubation at room temperature for 10 min in darkness, the absorbance was measured at 367 nm using a microplate reader. Results were expressed on a per-gram basis, which was calculated based on the weight of the sample used for the extraction. Quercetin was used as a standard to create a calibration curve, and results were expressed as milligrams of quercetin equivalents per gram of sample (mg QE/g).

S1.1.3. Total carotenoid content (TCC)

For the TCC assay, the method of [3] was used. Each sample: free-conventional, free-eutectic, and bound phytochemicals from PPBP and PPDF, was mixed in a ratio 1:10 with a solution of acetone/hexane (2:3 ratio), vortexed for 1 min, and pelleted for 10 min at 2000xg. The supernatant was transferred to a 96-well plate. The absorbance was read at 450 nm using a microplate reader. Results were expressed on a per-gram basis, which was calculated based on the weight of the sample used for the extraction. β-carotene was used as a standard to create a calibration curve, and results were expressed as milligrams of β-carotene equivalents per gram of sample (mg βCE/g).

S1.1.4. Total tannin content (TTC)

TTC were determined for free-conventional, free-eutectic, and bound phytochemicals from PPBP and PPDF, according to [4]. Briefly, a volume of each sample, was diluted

in water (1:10). Two aliquots were taken from each sample to two different tubes. In each tube, 1 mL of the sample was mixed with 0.5 mL of distilled water and 3 mL of 12 N HCl. Each aliquot from the same sample was subjected to a different treatment. The first aliquot was heated at 100°C for 30 min, while the second aliquot was kept at room temperature for the same period. After 30 min, the tubes from the first treatment were cooled. Then, 0.5 mL of absolute ethanol was added to each tube of the two aliquots to stop the reaction. Finally, samples were transferred to a 96-well plate and the absorbance recorded at 550 nm. Results were expressed on a per-gram basis, which was calculated based on the weight of the sample used for the extraction. Results were express in mg of cyanidin-3 glucoside equivalents per gram of sample (mgC3GE/g).

S1.2. Antioxidant Activity

The antioxidant activity of each sample: free-conventional, free-eutectic, and bound phytochemicals from PPBP and PPDF, was determined. For DPPH•, a volume of each sample was diluted in methanol; then, 20 μL of the diluted sample was mixed with 180 μL of 0.2 mM of DPPH• solution in methanol in a 96-well plate. The mixture was shaken and left to react for 15 min in the dark at room temperature. The absorbance of the reaction was measured at the beginning and at the end of the reaction in a microplate reader at 517 nm, according to [5]. For $ABTS^{•+}$ assays, 20 μL of the diluted samples were mixed with 180 μL of $ABTS^{•+}$ solution in ethanol in a 96-well plate. The mixture was incubated for 10 min at room temperature in the dark. The absorbance of the reaction was measured at the beginning and at the end of incubation in a microplate reader at 734 nm [6]. In both cases, the percentage of inhibition of the free radicals was calculated based on the absorbance values of the control and the sample. Results were expressed on a per-gram basis, which was calculated based on the weight of the sample used for the extraction. Results were expressed in mg of Trolox equivalent per gram of sample (mg TE/g).

S1.3. Dietary Fibre Determination

The method aimed to determine the amount of dietary fibre in a sample by differentiating between acid detergent fibre (ADF) and neutral detergent fibre (NDF) [7-9], being indicators of the ultrasonic-NADES treatment. For ADF extraction, 0.5 g of dried PPBP or PPDF was mixed with 50 mL of 1 N H2SO4 and 2% (w/v) of cetyltrimethylammonium bromide acid detergent. The solution was boiled for 1 h, the solid fraction filtered, washed, dried, and weighed to calculate the percentage of ADF. For NDF extraction, 0.5 g of PPBP or PPDF was mixed with 100 mL of a solution of 1.86% of EDTA, 0.68% of Na2B4O2, 1% of ethylene glycol, and 3% of sodium sulphite solution. The solution was boiled for 1 h. The resulting mixture was filtered, dried, and weighed to calculate the percentage of NDF.

S1.4. Multiple reaction monitoring (MRM) conditions of authentic standards

The optimized conditions of monosaccharide analysis by MRM is in Table S1.

Table S1. MRM conditions of monosaccharide identification

Monosaccharide	Retention time (min)	[PMP+M+H]+	MS2 [PMP + H]	CE/V
D-Manose	13.83	511.15	175	-35
Galacturonic acid	15.18	525.15	175	-35
D-Rhamnose	16.33	495.20	175	-35
D-Glucose	19.06	511.15	175	-35
D-Galactose	19.94	511.15	175	-35
Arabinose	21.04	481.20	175	-35
L-Fucose	22.88	495.20	175	-35

References

1. Singleton, V.L.; Orthofer, R.; Lamuela-Raventós, R.M. Analysis of total phenols and other oxidation substrates and antioxidants by means of folin-ciocalteu reagent. In *Methods in enzymology*; 1999; Volume 299, pp. 152-178.
2. Pękal, A.; Pyrzynska, K. Evaluation of Aluminium Complexation Reaction for Flavonoid Content Assay. *Food Analytical Methods volume* **2014**, *7*.
3. Nagata, M.; Yamashita, I. Simple method for simultaneous determination of chlorophyll and carotenoids in tomato fruit. *Nippon Shokuhin Kogyo Gakkaishi* **1992**, *39*, 925-928, doi:10.3136/nskkk1962.39.925.
4. Ribéreau-Gayon, P.; Glories, Y.; Maujean, A.; Dubourdieu, D. Phenolic compounds. In *Handbook of Enology: The Chemistry of Wine Stabilization and Treatments*, Sons, J.W., Ed.; Wiley: 2006; Volume 2, p. 161.
5. Brand-Williams, W.; Cuvelier, M.E.; Berset, C. Use of a free radical method to evaluate antioxidant activity. *LWT - Food Science and Technology* **1995**, *28*, 25-30.
6. Re, R.; Pellegrini, N.; Proteggente, A.; Pannala, A.; Yang, M.; Rice-Evans, C. Antioxidant activity applying an improved ABTS radical cation decolorization assay. *Free Radical Biology and Medicine* **1999**, *26*, 1231-1237, doi:10.1016/s0891-5849(98)00315-3.
7. Van Soest, P.J.; Robertson, J.B.; Lewis, B.A. Methods for dietary fiber, neutral detergent fiber, and nonstarch polysaccharides in relation to animal nutrition. *J Dairy Sci* **1991**, *74*, 3583-3597, doi:10.3168/jds.S0022-0302(91)78551-2.
8. Mertens, D.R. Gravimetric determination of amylase-treated neutral detergent fiber in feeds with refluxing in beakers or crucibles: collaborative study. *J AOAC Int* **2002**, *85*, 1217-1240.
9. Martí, N.; Saura, D.; Fuentes, E.; Lizama, V.; García, E.; Mico-Ballestera, M.J.; Lorente, J. Fiber from tangerine juice industry. *Industrial Crops and Products* **2011**, *33*. https://doi.org/10.1016/j.indcrop.2010.09.004
